# Salivary Microbiome Differences in Stunted and Healthy Children: A Metagenomic Analysis

**DOI:** 10.1055/s-0045-1814094

**Published:** 2026-01-20

**Authors:** Nila Kasuma, Haria Fitri, Reno Wiska Wulandari, Gian Ernesto, Dinda Ratna Juwita, Muhammad Dzaky Sayyid Effendi, Thifla Rafifa Wirza

**Affiliations:** 1 Department of Oral Biology, Faculty of Dentistry, Universitas Andalas, Padang, Sumatera Barat, Indonesia; 2 Department of Oral and Maxillofacial Surgery, Faculty of Dentistry, Universitas Andalas, Padang, Sumatera Barat, Indonesia; 3 Faculty of Dentistry, Universitas Andalas, Padang, Sumatera Barat, Indonesia

**Keywords:** stunting, salivary microbiome, 16S rRNA, nutritional status, oral dysbiosis, pediatric microbiota

## Abstract

**Objectives:**

This study aimed to compare the composition and diversity of the salivary microbiome in stunted and nonstunted children using 16S rRNA gene sequencing to explore the relationship between nutritional status and oral microbiota.

**Materials and Methods:**

A total of 20 saliva samples were collected from children aged 6 to 10 years, comprising two groups: stunted ( *n* = 10) and healthy controls ( *n* = 10). Deoxyribonucleic acid was extracted, and the V3–V4 region of the 16S rRNA gene was amplified and sequenced. Bioinformatics analysis included taxonomic assignment, calculation of relative abundance, α diversity (using Shannon and Simpson indices), β diversity (UniFrac-based principal coordinate analysis and permutational multivariate analysis of variance [PERMANOVA]), and differential abundance testing using the Mann–Whitney *U* test.

**Results:**

The dominant phyla in both groups were *Proteobacteria* , *Firmicutes* , and *Bacteroidota* , with *Proteobacteria* being more prevalent in the stunted group. At the genus level, *Neisseria* and *Veillonella* were more abundant in stunted children. Notably, *Veillonella* was significantly elevated in the stunted group (28.6%) compared with controls (14.9%, *p* = 0.0376). Alpha diversity indices revealed a higher diversity trend in the stunted group, although this difference was not statistically significant (Shannon, *p* = 0.130; Simpson, *p* = 0.762). Beta diversity analysis revealed no considerable clustering between groups (PERMANOVA *p* > 0.05), indicating moderate interindividual variability but no clear group separation.

**Conclusion:**

Children with stunted growth demonstrated distinct microbial signatures in their salivary microbiota, particularly in the increased abundance of *Proteobacteria* and *Veillonella* , suggesting a potential link between chronic undernutrition and oral microbial dysbiosis. These findings underscore the need for additional studies to investigate the impact of nutritional status on oral and systemic health through the microbiome axis.

## Introduction

Stunting remains a significant global health concern, affecting approximately 150.2 million children under the age of 5, or 23.2% of the worldwide population, as of 2024. 1 In Indonesia, despite extensive public health efforts, the prevalence of stunting remains high at 19.8%, exceeding the national target of 14% set for 2024. Stunting is defined by the World Health Organization (WHO) as a height-for-age z-score (HAZ) of less than –2 standard deviations (SDs), primarily resulting from chronic malnutrition and recurrent infections during the critical window of the first 1,000 days of life. 2 Contributing factors include low socioeconomic status, maternal undernutrition, poor sanitation, and inappropriate infant feeding practices, all of which may have long-term consequences on physical growth, cognitive function, school achievement, and adult economic productivity. 3

Beyond its impact on growth, stunting is increasingly associated with systemic immune dysregulation, placing affected children at higher risk for noncommunicable diseases such as hypertension, cardiovascular disease, and type 2 diabetes. 4 Furthermore, oral health complications are common among stunted children. Studies have reported increased prevalence of caries and gingivitis, attributed to low salivary flow rate, reduced buffering capacity, and dysfunction in salivary proteins, which in turn promote acidic environments that support pathogenic bacterial colonization. 5 These oral alterations may also reflect or contribute to systemic dysbiosis, particularly given the interplay between the oral and gut microbiota. 6

A growing body of literature highlights that children with stunting exhibit gut microbiome immaturity, characterized by reduced microbial diversity and a predominance of inflammatory or pathogenic taxa, which impairs nutrient synthesis and absorption. 7 The oral cavity, hosting the second-largest microbial community after the gut, is a crucial yet often overlooked site in this context. 8 Comprising bacteria, fungi, viruses, and protozoa, the oral microbiome plays a fundamental role in digestion, immunity, and maintaining oral-systemic health homeostasis. Disruption of this microbial balance (oral dysbiosis) can contribute not only to local disease but also to systemic inflammatory conditions. 9

Recent studies propose the oral–gut microbiome axis, a bidirectional pathway through which oral pathogens such as *Porphyromonas gingivalis* may translocate to the gastrointestinal tract, exacerbate inflammation, and modulate the gut microbial ecosystem. 10 This mechanism is particularly relevant for stunted children, who are more vulnerable to oral inflammation, and whose nutrient absorption may be further impaired by the cascading effects of dysbiosis across both sites. 11 These findings underscore the systemic implications of oral microbiome health in malnourished populations.

Despite growing interest in the microbiota's role in childhood stunting, most research has centered on the gut microbiome, with limited attention to the oral microbial community. 12 This study addresses a critical gap by characterizing the salivary microbiome profile in stunted children, an area that remains poorly understood. Unlike previous studies, this study characterizes the salivary microbiome in stunted children using 16S rRNA metagenomic sequencing. 13 This research aims to compare the salivary microbiota between stunted and nonstunted children at both phylum and genus levels, and to assess variations in α and β diversity. The central hypothesis is that chronic undernutrition in stunted children leads to significant dysbiosis, characterized by an increased abundance of specific taxa, such as *Proteobacteria* , *Spirochaetota* , and *Veillonella* , and disrupts the overall microbial community structure, potentially affecting both oral and systemic health outcomes.

## Material and Methods

### Study Design

An observational study was conducted to investigate and compare the composition and diversity of the salivary microbiome between stunted and healthy children. The study population consisted of children aged 6 to 12 years, selected based on specific inclusion criteria and informed consent from their parents. Children with diagnosed psychological or cognitive developmental disorders (e.g., autism spectrum disorder, attention deficit hyperactivity disorder, intellectual disability) were also excluded to avoid behavioral and neurocognitive confounding factors. A total of 20 subjects were enrolled and divided equally into two groups: stunted ( *n* = 10) and healthy controls ( *n* = 10).

### Sampling and Data Collection

Stunting classification was determined according to the WHO child growth standard using the HAZ. Height measurements were taken using a portable stadiometer with the children standing on a flat surface, and stunting was defined as a height below 2 SDs of the HAZ. Saliva samples were collected unstimulated from all participants using a sterile funnel into microtubes between 08:00 and 11:00 a.m. to control for circadian variation. Participants were instructed to refrain from eating, drinking, and brushing their teeth for at least 1 hour before sampling. All collected samples were stored at –80°C until further analysis. 14

### Metagenomic Sequencing

Microbial deoxyribonucleic acid (DNA) was extracted from saliva samples using the ZymoBIOMICS DNA Miniprep Kit. DNA quality and concentration were assessed through agarose gel electrophoresis. The V3–V4 region of the 16S rRNA gene was amplified using specific primers (forward primer: 5′-TCGTCGGCAGCGTCAGATGTGTATAAGAGACAGCCTACGGGNGGCWGCAG; reverse primer: 5′-GTCTCGTGGGCTCGGAGATGTGTATAAGAGACAGGACTACHVGGGTATCTAATC) and Q5 High-Fidelity DNA Polymerase (New England Biolabs). Polymerase chain reaction (PCR) products were visualized on agarose gel, purified using the Qiagen Gel Extraction Kit, and subjected to library preparation with the NEBNext Ultra DNA Library Prep Kit for Illumina. Barcode sequences were added through PCR indexing. A second purification was performed to remove remaining contaminants. The prepared libraries were quantified, normalized, pooled, and sequenced using the Illumina MiSeq platform with a 2 × 250 base pair paired-end protocol. Raw sequencing data were processed using MiSeq Reporter and QIIME2 software for primer trimming, quality filtering, and microbial analysis, including taxonomic profiling and diversity estimation. Operational taxonomic units were clustered at 97% similarity, and taxonomic annotation was performed using the SILVA 16S rRNA database version 138.1 ( http://ngs.arb-silva.de/ ) 15 .

### Diversity Analysis

Microbial diversity was evaluated through both α and β diversity indices. Alpha diversity, representing within-sample diversity, was calculated using the Shannon index (richness) and Simpson index (evenness). Beta diversity, representing between-group diversity, was analyzed using unweighted and weighted UniFrac distance metrics. Principal coordinate analysis (PCoA) was conducted to visualize differences in community structure among the two groups.

### Statistical Analysis

Statistical comparisons of microbial diversity and composition between groups were performed using R software. The Mann–Whitney *U* test was used to assess differences in the relative abundances of bacterial taxa at both the phylum and genus levels. Alpha diversity indices were compared using the Kruskal–Wallis test, and β diversity was evaluated using permutational multivariate analysis of variance (PERMANOVA). A *p* -value of < 0.05 was considered statistically significant. No multiple-testing correction was applied, given the exploratory and pilot nature of the study.

### Ethical Considerations

This study was conducted in accordance with the principles of the Declaration of Helsinki and received approval from the Ethics Committee of the Faculty of Medicine, Universitas Andalas, Number 291/UN.16.2/KEP-FK/2024. Informed consent was obtained from all parents or legal guardians of the participating children.

## Results

Table 1 summarizes the characteristics of two participant groups: stunted children and healthy controls, each with 10 subjects. Both groups had similar mean ages (8.4 vs. 8.6 years) and balanced sex distributions, minimizing demographic confounders. The body mass index (BMI) was significantly lower in the stunted group (mean: 13.5 kg/m ^2^ ) compared with the control group (mean: 16.8 kg/m ^2^ ), confirming their classification based on WHO stunting criteria. These differences suggest a notable nutritional disparity, which may contribute to the distinct salivary microbiome profiles observed between the two groups.

**Table 1:** Subject characteristics based on age, sex, and BMI

Characteristic	Stunted group ( *n* = 10)	%	Control group ( *n* = 10)	%
Age (y)				
• Mean	8.4	—	8.6	—
• Median	8.5	—	8.8	—
• Range	6.5–10.2	—	6.7–10.4	—
Sex				
• Male	6	60	5	50
• Female	4	40	5	50
BMI (kg/m ^2^ )				
• Mean	13.5	—	16.8	—
• Median	13.6	—	16.9	—
• Range	12.4–14.3	—	15.9–17.6	—

Abbreviation: BMI, body mass index.

Note: Data source is the author'sown research.

Fig. 1 compares the salivary microbiota profiles of stunted and nonstunted children at both the phylum and genus levels. The stunted group exhibited a dominance of *Proteobacteria* and *Neisseria* , while the control group had higher levels of *Firmicutes* , *Streptococcus* , *Veillonella* , and *Prevotella* . These microbial differences suggest that chronic undernutrition may lead to oral microbiome dysbiosis, which could impact both oral and systemic health. The findings highlight the influence of nutritional status on microbial diversity, suggesting that further investigation is warranted into its broader health implications.

**Fig. 1:**
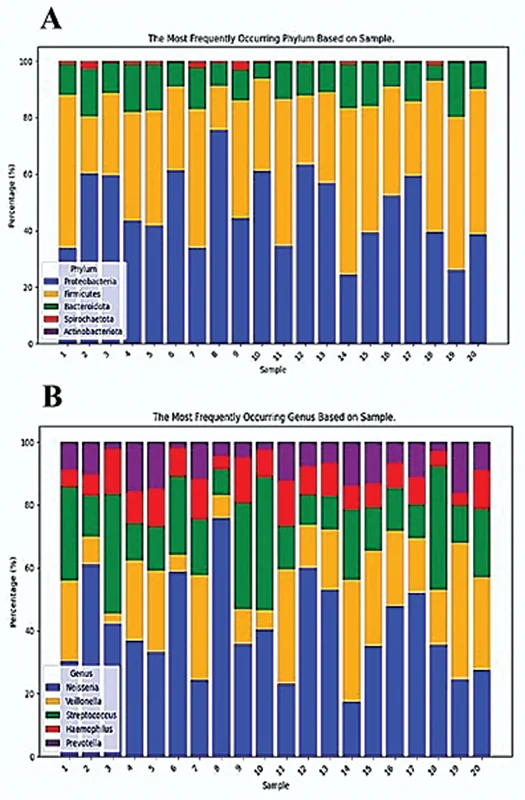
Top 5 phylum microbial composition ( **A** ) and genera ( **B** ) composition based on sample (stunted samples 1–10 and control samples 11–20). This study shows that the most abundant phyla are *Proteobacteria* , *Firmicutes* , *Bacteroidota* , *Spirochaetota* , and *Actinobacteriota* , respectively ( **A** ). Meanwhile, *Neisseria* , *Veillonella* , *Streptococcus* , *Haemophilus* , and *Prevotella* are the top 5 genera ( **B** ).

Fig. 2 illustrates the relative abundance of salivary microbiota between stunted and nonstunted children. At the phylum level, both groups were dominated by *Proteobacteria* , *Firmicutes* , and *Bacteroidota* , with *Proteobacteria* being more abundant in the stunted group and *Firmicutes* relatively higher in controls. A statistically significant increase in *Spirochaetota* was also observed among the stunted group ( *p* = 0.0189). At the genus level, *Neisseria* was the most dominant in both groups, followed by *Veillonella* and *Streptococcus* . Notably, *Veillonella* exhibited a significantly higher abundance in the stunted group (28.6% vs. 14.9%, *p* = 0.0376), while *Streptococcus* was higher in stunted children but without statistical significance. These microbial patterns suggest that specific taxa, particularly *Veillonella* and *Spirochaetota* , may be associated with stunting through mechanisms related to immune modulation or nutrient metabolism.

**Fig. 2:**
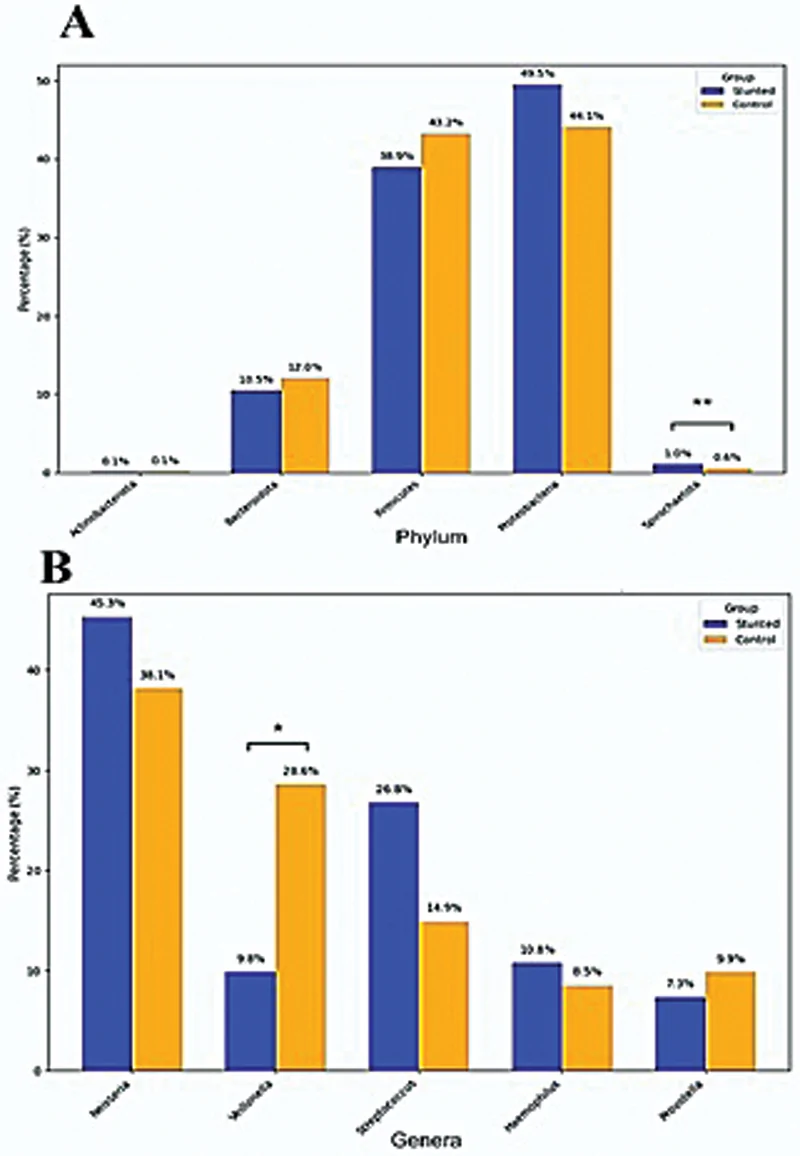
Relative abundance comparison of major bacterial phyla ( **A** ) and genera ( **B** ) between stunted and control groups. Proteobacteria was more abundant in the stunted group, while *Firmicutes* predominated in controls. At the genus level, *Veillonella* showed significantly higher abundance in the stunted children ( *p* < 0.05).

The comparison of α diversity between the stunted and control groups is illustrated in Fig. 3 , which utilizes the Shannon index ( Fig. 3A ) and the Simpson's index ( Fig. 3B ) to evaluate microbial richness and evenness within each group. In the Shannon index, the stunted group showed a trend toward higher microbial diversity, characterized by a broader distribution and higher central tendency values compared with the control group. Similarly, the Simpson's index also revealed slightly greater diversity in the stunted group, though less prominently. Despite these observed trends, statistical analysis using the Kruskal–Wallis test showed that neither of the differences was statistically significant, with *p* -values of 0.130 for the Shannon index and 0.762 for the Simpson's index. These results suggest that although a potential increase in microbial diversity may be associated with stunting, the difference is not robust enough to conclude a significant alteration in overall salivary microbial diversity between stunted and healthy children. This nonsignificant difference might be attributed to the small sample size or natural interindividual variability in the oral microbiota. Nonetheless, the observed patterns warrant further investigation, particularly in relation to how nutritional status might influence the microbial ecology of the oral cavity.

**Fig. 3:**
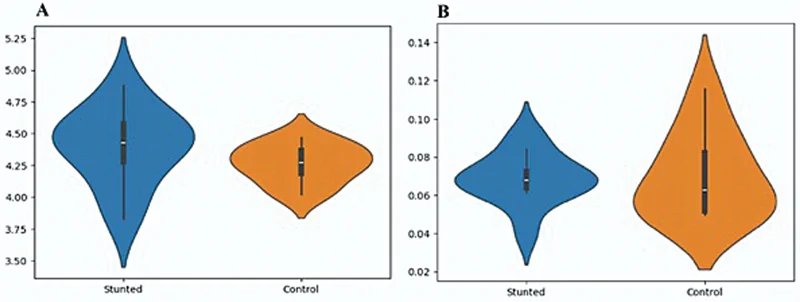
Alpha diversity comparisons of the stunted and control groups. Shannon ( **A** ) and Simpson's index ( **B** ) significance were analyzed with Kruskal–Wallis. Significance determined by *p* < 0.05 (*), ≤ 0.01 (**), ≤ 0.001 (***).

Fig. 4 illustrates β diversity comparisons between stunted and nonstunted (control) children using PCoA based on UniFrac distances. Both unweighted (panel A) and weighted (panel B) analyses show overlapping distributions between groups. While axis 1 explains up to 26.41% of the variance in the weighted plot, no distinct clustering or separation between groups is observed. This indicates that stunting does not significantly impact the overall community structure of the salivary microbiota, suggesting a high degree of microbial community similarity despite nutritional status differences.

**Fig. 4:**
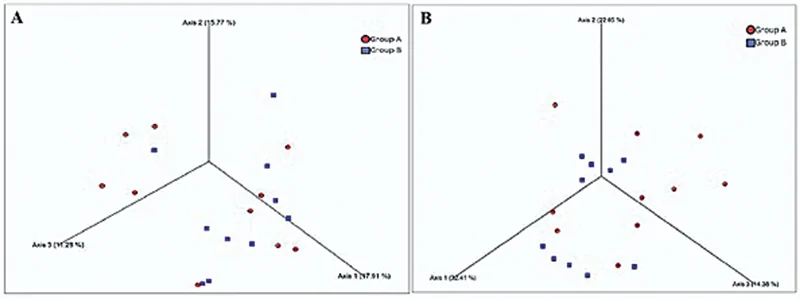
Beta diversity between stunted group and control group in ( **A** ) unweighted and ( **B** ) weighted UniFrac metrics.

Fig. 5 compares β diversity between stunted and control children using unweighted and weighted UniFrac metrics. Although the stunted group showed slightly higher average distances to the centroid, indicating greater microbiome variation, the differences were not statistically significant (PERMANOVA: *p* > 0.05). The unweighted distance (0.48 vs. 0.39) and weighted distance (0.27 vs. 0.21) suggest a trend of higher heterogeneity in the stunted group, possibly due to varied nutritional or environmental exposures. However, overall community composition remained comparable between groups.

**Fig. 5:**
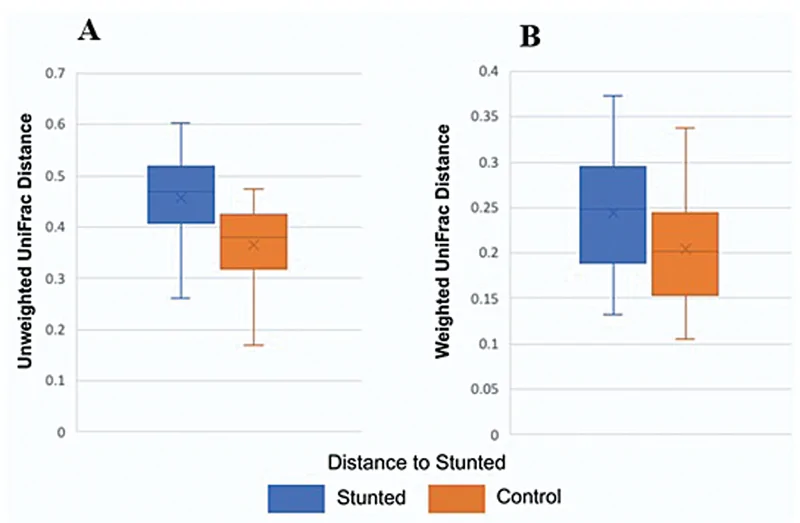
Beta diversity comparisons between the stunted and the control groups. Unweighted ( **A** ) and weighted ( **B** ) UniFrac stunted distance comparisons using permutational multivariate analysis of variance (PERMANOVA). Significance determined by *p* < 0.05 (*), ≤0.01 (**), ≤ 0.001 (***).

## Discussion

The findings of this study offer an initial insight into the differences in salivary microbiome profiles between stunted children and their healthy counterparts. Although no statistically significant differences were observed in α and β diversity indices, taxonomic analysis revealed tendencies toward oral microbial dysbiosis in the stunted group. This suggests that nutritional status may play a role in shaping the oral microbiota composition, potentially influencing both oral and systemic health outcomes. These results underscore the importance of investigating the microbiome as a potential biomarker or contributing factor in growth disorders. The following discussion explores the biological implications of these findings, acknowledges the study's limitations, and outlines future research directions for more comprehensive and longitudinal analyses.

This study revealed a significant difference in BMI between the stunted and control groups, with stunted children exhibiting a significantly lower average BMI (13.5 kg/m ^2^ ) compared with the control group (16.8 kg/m ^2^ ). These results align with the WHO classification of stunting, characterized by chronic undernutrition and impaired linear growth. 16 Malnutrition can impact systemic health and oral immunity, disrupting the balance of the oral microbiome. Nutritional deficiencies, such as those of vitamin D and zinc, are known to impair mucosal defenses and alter oral microbial composition. 17

At the taxonomic level, both the stunted and control groups exhibited dominance by five major phyla: *Proteobacteria* , *Firmicutes* , *Bacteroidota* , *Spirochaetota* , and *Actinobacteriota* . The most prevalent genera were *Neisseria* , *Veillonella* , *Streptococcus* , *Haemophilus* , and *Prevotella* . These findings are consistent with the oral microbiome profiles reported in healthy populations. 18 However, the increased proportion of *Veillonella* in the stunted group may indicate a shift toward a more acidogenic and potentially dysbiotic environment, as *Veillonella* thrives in acidic niches formed by carbohydrate metabolism. 19

Quantitative analysis revealed that *Veillonella* (at the genus level) and *Spirochaetota* (at the phylum level) were significantly more abundant in the stunted group compared with controls ( *p* < 0.05). *Veillonella* abundance nearly doubled (28.6% vs. 14.9%) in stunted children, suggesting an oral environment favoring acid-tolerant species and possibly early signs of dysbiosis. 20 The increased presence of *Spirochaetota* , which includes pathogenic species such as *Treponema* , could be associated with low-grade inflammation or weakened host immunity, and has been linked to chronic periodontitis. 10 These taxa may therefore represent early microbiome signatures associated with chronic undernutrition in children. However, the absence of multiple-testing correction may increase the likelihood of false-positive findings, and thus the statistically significant taxa reported here should be interpreted as exploratory rather than confirmatory. Taken together, these findings support the need for larger and longitudinal studies. These findings are consistent with previous reports showing an overrepresentation of acidogenic or inflammation-associated taxa in undernourished pediatric populations, particularly the enrichment of *Veillonella* in stunted cohorts reported in earlier studies. 21

Alpha diversity, as measured by the Shannon and Simpson indices, was higher in the stunted group; however, the difference was statistically insignificant ( *p* > 0.05). This suggests that while microbial richness and evenness may appear elevated, it does not reflect a consistent pattern of dysbiosis. Similar findings have been reported in other pediatric cohorts with growth faltering, where taxonomic reorganization rather than diversity reduction characterizes microbial changes. 22

PCoA of β diversity, using both unweighted and weighted UniFrac metrics, revealed interspersed clustering between the stunted and control groups. 23 This indicates that the overall community structure of the salivary microbiome did not segregate distinctly by nutritional status. A comparable pattern was observed in Bangladeshi children with growth stunting, suggesting that dysbiosis in stunting may be subtle and confined to specific taxa. 24

PERMANOVA analysis further confirmed the absence of significant differences in microbial community structure between the groups. Unweighted UniFrac yielded a pseudo- *F* value of 0.949 ( *p* = 0.535), and weighted UniFrac showed a pseudo- *F* of 1.876 ( *p* = 0.086), indicating no statistically significant dissimilarity. This suggests that the overall microbial architecture remains largely unchanged, even though the relative abundance of key taxa, such as *Veillonella* , varies. These findings align with prior studies from sub-Saharan Africa and India, which have also found that malnutrition does not significantly alter β diversity. 25

The elevated abundance of *Veillonella* and *Spirochaetota* in stunted children may serve as early biomarkers for oral microbial shifts associated with poor nutrition. 13 These findings support the potential use of salivary microbiome analysis as a noninvasive diagnostic tool for nutritional assessment. 13 This study holds significant potential for advancing scientific knowledge in the field of nutritional status and interactions between the oral microbiome and children. By identifying an increased abundance of specific genera, such as *Veillonella* , and phyla like *Spirochaetota* in stunted children, it provides novel insights into how malnutrition may shape the composition of the salivary microbiota.

These findings lay the groundwork for future research exploring the use of salivary microbiome profiles as noninvasive biomarkers for early detection and monitoring of stunting, and may open avenues for microbiome-targeted nutritional interventions, particularly in resource-limited settings where conventional diagnostic methods are challenging. Nevertheless, the present analysis was limited to salivary samples and did not include parallel profiling of the gut microbiome from the same cohort, which would have provided a more comprehensive understanding of the oral–gut axis in growth faltering. Future studies are therefore warranted to incorporate combined oral and gut metagenomic analyses to determine whether salivary microbial shifts reflect or precede gastrointestinal dysbiosis in stunted children.

Although this study provides novel insight into the salivary microbiome profile of stunted children, several limitations should be acknowledged. The small sample size ( *n* = 20) reduces statistical power and generalizability, and as such this work should be regarded as a pilot investigation. The cross-sectional design also precludes causal inference. Furthermore, the analysis was restricted to salivary microbiota without parallel assessment of gut microbiome or systemic biomarkers, limiting the interpretation of the oral–gut axis in chronic undernutrition. Future studies should employ longitudinal designs with integrated metagenomic and metabolomic approaches to better elucidate the functional and systemic consequences of microbiome alterations in growth-faltered children.

## Conclusion

This study highlights significant differences in the salivary microbiota composition between stunted and nonstunted children. At the phylum level, *Spirochaetota* was found to be significantly more abundant in the stunted group. At the genus level, *Veillonella* exhibited a notably higher relative abundance in stunted children compared with controls. Although the α diversity indices (Shannon and Simpson) suggested a trend of greater microbial diversity in the stunted group, these differences were not statistically significant. Furthermore, β diversity analysis revealed no distinct clustering between the two groups, indicating an overlapping microbial community structure. These findings suggest that poor nutritional status associated with stunting may influence specific microbial taxa in the oral cavity.

## References

[1] Apriliani A Utilization of primary healthcare services and stunting among children aged 0–59 months in Indonesia: a national cross-sectional study Contagion 2025 7 01 404 414

[2] Beal T Tumilowicz A Sutrisna A Izwardy D Neufeld L M A review of child stunting determinants in Indonesia Matern Child Nutr 2018 14 04 e12617 10.1111/mcn.12617 PMC6175423 29770565

[3] Lestari T RP Stunting in Indonesia: understanding the roots of the problem and solutions Info Singkat 2023 15 14 21 25

[4] Grey K Gonzales G B Abera M Severe malnutrition or famine exposure in childhood and cardiometabolic non-communicable disease later in life: a systematic review BMJ Glob Health 2021 6 03 e003161 10.1136/bmjgh-2020-003161 PMC7949429 33692144

[5] Vieira K A Rosa-Júnior L S Souza M AV Santos N B Florêncio T MMT Bussadori S K Chronic malnutrition and oral health status in children aged 1 to 5 years: an observational study Medicine (Baltimore) 2020 99 18 e19595 10.1097/MD.0000000000019595 PMC7440136 32358344

[6] Thomas C Minty M Vinel A Oral microbiota: a major player in the diagnosis of systemic diseases Diagnostics (Basel) 2021 11 08 1376 10.3390/diagnostics11081376 PMC8391932 34441309

[7] Ebrahim M Manji K Role of infant and early-childhood nutrition on gut inflammation, stunting, growth, and development in the African context: a narrative review Nutr Clin Pract 2025 40 03 534 542 10.1002/ncp.11270 39777420

[8] Kitamoto S Nagao-Kitamoto H Hein R Schmidt T M Kamada N The bacterial connection between the oral cavity and the gut diseases J Dent Res 2020 99 09 1021 1029 10.1177/0022034520924633 PMC7375741 32464078

[9] Chandra Nayak S Latha P B Kandanattu B Pympallil U Kumar A Kumar Banga H The oral microbiome and systemic health: bridging the gap between dentistry and medicine Cureus 2025 17 02 e78918 10.7759/cureus.78918 PMC11909285 40091996

[10] Zhou T Xu W Wang Q The effect of the “Oral-Gut” axis on periodontitis in inflammatory bowel disease: a review of microbe and immune mechanism associations Front Cell Infect Microbiol 2023 13 1.13242E6 10.3389/fcimb.2023.1132420 PMC10008960 36923589

[11] Vonaesch P Araújo J R Gody J-C Afribiota Investigators Stunted children display ectopic small intestinal colonization by oral bacteria, which cause lipid malabsorption in experimental models Proc Natl Acad Sci U S A 2022 119 41 e2209589119 10.1073/pnas.2209589119 PMC9573096 36197997

[12] Tjandrawinata R R Amalia N Tandi Y YP The forgotten link: how the oral microbiome shapes childhood growth and development Front Oral Health 2025 6 1.547099E6 10.3389/froh.2025.1547099 PMC11842321 39989601

[13] Octaricha T Ilmiawati C Kasuma N Salivary microbiome profile shifts after scaling in stunted children BMC Res Notes 2025 18 01 69 10.1186/s13104-025-07147-w PMC11830171 39956914

[14] Kasuma N Pratiwi R A Aulia R K Juwita D R Ernesto G Effectiveness of oil pulling with virgin coconut oil (VCO) on Streptococcus mutans count in saliva of stunted children J Int Dent Med Res 2025 18 02 821 827

[15] Chen Y Fan L C Chai Y H Xu J F Advantages and challenges of metagenomic sequencing for the diagnosis of pulmonary infectious diseases Clin Respir J 2022 16 10 646 656 10.1111/crj.13538 PMC9527156 36068680

[16] Al-Qerem W Zumot R Jarab A Eberhardt J Alasmari F Hammad A Prevalence of being obese, overweight, and underweight among Jordanian children and adolescents based on international growth standards Healthcare (Basel) 2025 13 02 146 10.3390/healthcare13020146 PMC11765480 39857173

[17] Morales F Montserrat-de la Paz S Leon M J Rivero-Pino F Effects of malnutrition on the immune system and infection and the role of nutritional strategies regarding improvements in children's health status: a literature review Nutrients 2023 16 01 1 10.3390/nu16010001 PMC10780435 38201831

[18] Widyarman A S Theodorea C F Udawatte N S Diversity of oral microbiome of women from urban and rural areas of Indonesia: a pilot study Front Oral Health 2021 2 738306 10.3389/froh.2021.738306 PMC8757682 35048055

[19] Wicaksono D P Washio J Abiko Y Domon H Takahashi N Nitrite production from nitrate and its link with lactate metabolism in oral *Veillonella* spp Appl Environ Microbiol 2020 86 20 e01255-20 10.1128/AEM.01255-20 PMC7531945 32769185

[20] Theodorea C F Diven S Hendrawan D Characterization of oral *Veillonella* species in dental biofilms in healthy and stunted groups of children aged 6-7 years in East Nusa Tenggara Int J Environ Res Public Health 2022 19 21 13998 10.3390/ijerph192113998 PMC9656475 36360876

[21] Collard J M Andrianonimiadana L Habib A Afribiota Investigators High prevalence of small intestine bacteria overgrowth and asymptomatic carriage of enteric pathogens in stunted children in Antananarivo, Madagascar PLoS Negl Trop Dis 2022 16 05 e0009849 10.1371/journal.pntd.0009849 PMC9119516 35533199

[22] Neves L L Hair A B Preidis G A A systematic review of associations between gut microbiota composition and growth failure in preterm neonates Gut Microbes 2023 15 01 2.190301E6 10.1080/19490976.2023.2190301 PMC10026866 36927287

[23] Chibuye M Mende D R Spijker R Systematic review of associations between gut microbiome composition and stunting in under-five children NPJ Biofilms Microbiomes 2024 10 01 46 10.1038/s41522-024-00517-5 PMC11116508 38782939

[24] Chehab R F Cross T L Forman M R The gut microbiota: a promising target in the relation between complementary feeding and child undernutrition Adv Nutr 2021 12 03 969 979 10.1093/advances/nmaa146 PMC8166545 33216115

[25] Govender P Ghai M Population-specific differences in the human microbiome: factors defining the diversity Gene 2025 933 148923 10.1016/j.gene.2024.148923 39244168

